# THE ANTHROPOCENE AND THE LONGEVITY REVOLUTION

**DOI:** 10.1093/geroni/igz038.1994

**Published:** 2019-11-08

**Authors:** Moon Choi

**Affiliations:** KAIST Graduate School of Science and Technology Policy, Daejeon, Korea, Republic of

## Abstract

The Anthropocene, a term popularized in 2010 by Nobel Prize-winning chemist Paul Crutzen, refers to the current epoch during which human beings have begun to have a significant impact on the earth, e.g., the environment and climate change. Global population has grown approximately seven-fold over the past 200 years, while average life expectancy at birth has dramatically increased due to improvements in nutrition, medicine, and technology. The human Longevity Revolution thus provides important evidence of the Anthropocene. Yet, in the face of the Anthropocene, contemporary lifestyles rooted in capitalism–continually seeking more and bigger–are not sustainable; changes are needed for humanity to “live long on the damaged planet.” This presentation will discuss the Longevity Revolution in the context of the theory and previous research on the Anthropocene, then suggest an agenda for future research related to the intersection between the Anthropocene and the Longevity Revolution.

